# Metastatic Latency, a Veiled Threat

**DOI:** 10.3389/fimmu.2019.01836

**Published:** 2019-08-06

**Authors:** Kangsan Kim, Mauricio Marquez-Palencia, Srinivas Malladi

**Affiliations:** ^1^Department of Pathology, UT Southwestern Medical Center, Dallas, TX, United States; ^2^Harold C. Simmons Comprehensive Cancer Center, UT Southwestern Medical Center, Dallas, TX, United States

**Keywords:** metastasis, dormancy, immune-surveillance, microenvironment, minimal residual disease, latency

## Abstract

Metastatic relapse is observed in cancer patients with no clinical evidence of disease for months to decades after initial diagnosis and treatment. Disseminated cancer cells that are capable of entering reversible cell cycle arrest are believed to be responsible for these late metastatic relapses. Dynamic interactions between the latent disseminated tumor cells and their surrounding microenvironment aid cancer cell survival and facilitate escape from immune surveillance. Here, we highlight findings from preclinical models that provide a conceptual framework to define and target the latent metastatic phase of tumor progression. The hope is by identifying patients harboring latent metastatic cells and providing therapeutic options to eliminate metastatic seeds prior to their emergence will result in long lasting cures.

## Introduction

In a significant number of cancer patients considered disease free, metastatic relapses occur. If and when relapse will occur is a question that is both indeterminate and unanswerable. Depending on the tumor type, these relapses might occur within a few months or decades after initial diagnosis and treatment (1–3). Very late recurrences are reported in a subset of breast cancer, head and neck squamous cell carcinoma (HNSCC), prostate cancer, melanoma and renal cell carcinoma patients considered disease free, presenting a major treatment follow-up challenge (4–18) (Table 1). In comparison, small cell lung cancer patients with aggressive disease have no reported latency as they are diagnosed with metastatic disease and have very poor survival rate (19). Lung cancers have short metastatic latency spans, with majority of relapses occurring within a year (20). Breast cancers with high proliferative index, triple negative breast cancers (TNBCs), tend to have shorter latency periods compared to estrogen receptor (ER) positive breast cancer (21). The frequency of late recurrence after 5 years is greatly reduced in TNBCs compared to ER^+^ tumors, where disease recurrences have been reported in a significant number of patients as late as 20 years after primary diagnosis (22–24). Human autopsy and transplant studies report existence of disseminated tumor cells (DTCs; tumor cells that extravasate and reside in secondary organs) or metastatic lesions that persist as occult disease, highlighting the role of host immune system in limiting metastatic outgrowth (25, 26). Latency competent cancer cells (LCCs) are slow cycling or quiescent DTCs that persist in organs after surgery and initial therapy, and are the major source of disease relapse (2, 3, 27). LCCs reside next to the vasculature and are surrounded by extracellular matrix (ECM), soluble factors, stromal, and immune cells. LCCs remain unscathed in these sanctuaries, undergoing genetic/epigenomic adaptations that augment their ability to initiate metastasis and impede immune surveillance. Metastatic latency therefore depends on the oncogenomic status of the disseminated tumor cells, their proliferative capacity and the surrounding microenvironment.

**Table 1:** Metastatic relapse rate and latency span in cancer patients.

**Cancer type**	**Late recurrence rate**	**Late relapse span**	**References**
Breast	~15–20%	1–22 years	(4, 13, 14, 21, 23, 24)
Prostate	~9.7–44%	1–20 years	(9, 13, 15)
Melanoma	~6.8–11.3%	15–20 years	(7, 12, 18)
Renal	~11–40%	1–25 years	(8, 13)
Lung	~10–24%	Months–5 years	(13, 20)
Head and neck	~24–33%	1–4 years	(13, 17)

Given that metastasis is the major cause for mortality in cancer patients, understanding how DTCs stay quiescent and remain viable for years before initiating metastasis is very critical (28). Assays to monitor the elusive LCCs and treatment strategies to effectively restrain or eliminate residual cancer cells is an unmet clinical need. Incorporating oncogenomic features of these cells along with tumor staging, presence of circulating/disseminated tumor cells or cell-free tumor DNA, will lead to better prediction of disease relapse in cancer patients with occult disease. Here, we summarize key determinants of metastatic latency, current concepts and proposed strategies to target and eliminate residual disease.

## Dissemination: Get Out of Dodge

As solid tumors grow, tissue constraints and cellular energy needs drive genetic or epigenetic changes in cancer cells that facilitate epithelial to mesenchymal transition and acquisition of invasive and stem-cell like characteristics (29–32). Key concepts discussing dormancy and epithelial mesenchymal transition (EMT) have been recently reviewed (33). Invasive cancer cells within the primary tumors breach the basement membrane, permeate the surrounding tissue as single cells or clumps and migrate into vasculature or lymphatics (31, 34). How and when tumors become invasive in patients? Are the early or late disseminated tumor cells from the primary tumor responsible for initiating metastasis in patients (35, 36)? Do the early disseminators aid in developing pre-metastatic niche (37, 38)? And the role of stromal cells in driving invasion (39), are some of the open questions actively investigated.

Although cancer cells intravasate in large numbers, very few survive in circulation. Given their prognostic value, circulating tumor cell (CTC) counts have been used to predict relapse or metastatic disease in breast, colorectal, small cell lung, and prostate cancer patients (40–43). Efforts from several labs have been directed toward improving CTC capture and enrichment protocols to define surface biomarkers on these potential metastatic seeds and to predict metastatic incidence (44, 45). Many ultrasensitive devices that are able to segregate CTCs from patient blood using size, density, electrical and compressibility differences have been developed to address this clinical need (46–50). However, isolating viable CTCs and performing functional experiments has been a challenge. With improved protocols and devices, several groups are now able to isolate, culture and characterize CTCs from patients (51–53). Such models are indispensable to study and advance concepts in metastatic evolution (44). CTC clusters or aggregates have also been isolated from blood stream and are reported to have greater predisposition to form metastasis than single cells in animal models (54). How CTC clusters survive the shear stress in circulation and avoid entrapment in lung capillaries is unclear (55). Further research is needed to determine how CTC clusters are dispersed or assembled in circulation (56, 57); what are they composed of; and what aspect of clustering aids metastatic competence. It should be noted that reliable CTC isolation and characterization is feasible only in metastatic disease and may not be able to identify patients with minimal residual disease. Nonetheless, diagnostic leukapheresis may enable reliable detection of CTCs in non-metastatic patients (58, 59).

## Metastatic Latency: It's not Kansas Anymore to Make Oneself at Home

**Figure 1:**
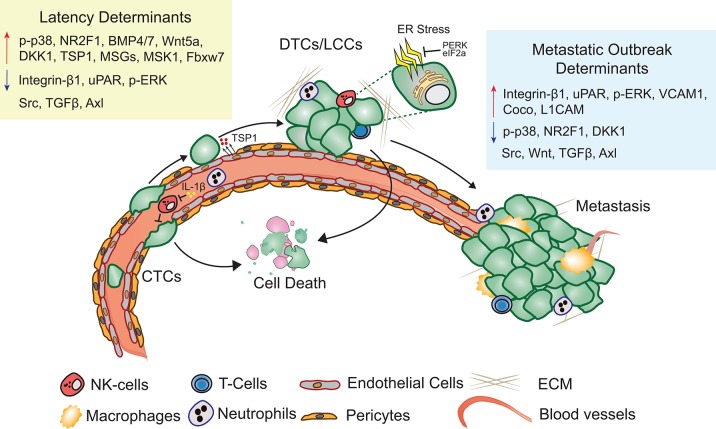
Metastatic latency. Upon extravasation, many DTCs perish, few surviving LCCs adapt to and modify the surrounding microenvironment, eventually giving rise to metastasis. Key molecular determinants of latency and metastatic outbreak are highlighted (Refer text for more details).

Metastatic latency span is both variable and indeterminate as it is a function of the rate at which the disseminated cancer cells adapt to and alter the surrounding microenvironment to initiate a metastatic lesion that impairs organ function. The composition and architecture of metastatic microenvironment determines the likelihood of DTC colonization (60). Majority of CTCs that extravasate into the new cellular milieu face resistance and perish upon extravasation (61). Cancer cells therefore have the propensity to reside in precincts that resemble stroma of the primary tumor (62). Depending on the robustness of the perceived cues, cancer cells are likely to proliferate, apoptose, or enter into a quiescent-slow cycling state (Figure 1). Proliferating DTCs are also more likely to be eliminated by chemotherapies and adjuvant therapies (22, 63). Slow cycling and quiescent LCCs, that are adapting to the new microenvironment remain unaffected by therapies targeting dividing cells and are enriched for stem cell like characteristics, that are critical to initiate secondary or metastatic tumors (27, 64, 65). Hypoxic microenvironments in the primary tumors promotes activation of dormancy programs and DTCs with these features are likely to survive better post-extravasation (66). Overall, absence of proliferating signals or a self-imposed block to these cues may result in activation of dormancy programs (27).

### Extracellular Matrix

The distribution and availability of growth factors and cytokines is tightly regulated by the ECM surrounding DTCs (37, 67). Non-structural matrix protein such as Thrombospondin-1 (TSP-1) and Periostin through direct interaction with membrane receptors and fibrous ECM molecules modulate cancer cell proliferation status (68). Collagen enriched fibrotic environment leads to activation of myosin light chain kinase through integrin β1 signaling and promotes proliferation in cancer cells, while failure to engage proliferative signals results in dormancy (69, 70). Mitogen activated protein kinase (MAPK) activity has been clearly demonstrated to regulate proliferation status of human squamous carcinoma, melanoma, breast, and prostate cancer cells (71). Increased p38 and decreased ERK activity is observed in dormant cancer cells. Urokinase-type plasminogen activator receptor (uPAR) drives activation of ERK through FAK and Src kinases and promotes proliferation, while loss of either uPAR expression or FAK/Src activity leads to increased p38 kinase activity and unleashes downstream quiescence effectors DEC2, NR2F1, and CDK inhibitors (60, 64, 72–74). Src activation in response to CXCL12 and IGF1, potentiates PI3K/AKT activation, and aids survival of latent breast cancer DTCs independent of their hormone receptor status or cancer subtype (62). Expression of metastatic suppressor genes (MSGs: KISS1, KAI1, MKK4/6/7, and NM23) has also been reported to limit metastasis initiating capacity of DTCs by modulating the activity of MAPKs through G-protein coupled receptors and tyrosine receptor kinases (75, 76). Over-expression of KISS1 results in limiting metastatic outgrowth of aggressive human melanoma cell line (77). Similarly, NM23 and MKK4/6 activate p38 and inhibit ERK to induce dormancy in ovarian and breast cancer cells (76). Mitogen and stress-activated kinase 1 (MSK1) functions downstream of p38 and restrains breast DTCs into a steady micro-metastatic state by promoting luminal differentiation through GATA3 and FOXA1 in ER^+^ breast cancer (78). Along these lines, GATA6 and HOPX have been reported to limit lung adenocarcinoma metastasis by promoting alveolar differentiation (79). Integrin α5β3 signaling response can also promote differentiation of luminal A breast cancer cells and limit tumor progression (80). L1CAM and YAP signaling enable the outgrowth of metastasis-initiating cells through integrin-ILK both immediately following their infiltration of target organs and as they exit metastatic latency (81). Taken together, altered ECM and MAPK activity in response to microenvironmental cues influences the proliferation status of latent DTCs.

### Endoplasmic Reticulum (ER) Stress

Transducers of unfolded protein response (PERK and eIF2α) are also activated in p38 active dormant cells and have been shown to be essential for cancer cell survival under chemotherapy induced genotoxic stress (74, 82). CK19 and MHC class I negative dormant pancreatic DTCs activate PERK and relieving ER stress pharmacologically or by expression of XBP1 in combination with T-cell depletion resulted in metastatic outgrowth (83). Administration of chemical chaperone 4-PBA to relieve ER stress in DTCs preoperatively has been proposed to drive DTCs out of quiescence and be cleared by active adaptive immune surveillance (83). Likewise, Fbxw7, a component of SCF-E3 ubiquitin ligase complex has been reported to maintain dormancy in breast DTCs and its ablation led to increased proliferation in this model system. A combination of depleting Fbxw7 and chemotherapy has been proposed to limit residual disease (84). Will these approaches result in reduced metastatic incidence or worsen survival outcome in patients by unleashing restrained heterogeneous metastatic clones needs to be further explored.

### Supportive Niches

Specialized microenvironments surrounding LCCs limit proliferation and facilitate cancer cell survival and quiescence. For example, perivascular niche (PVN) supports survival of hematopoietic stem cells (HSCs) as well as disseminated lung, melanoma, breast and prostate cancer cells in the bone marrow (85–87). TSP1 secreted by the microvascular endothelium in the bone and lung induce growth arrest in breast DTCs, while high Periostin and TGF-β1 expression in the neovascular tip cells triggers metastatic relapse (68). Inhibition of integrin-mediated interactions between DTCs (either quiescent or proliferating) and PVN sensitizes them to chemotherapy (88). Several stromal derived factors have inhibitory effect on LCC proliferation. For example, leukemia inhibitory factor (LIF) secreted by osteoblasts and bone marrow stromal cells limit growth of breast cancer DTCs in bone by activating LIFR: STAT3 signaling (89). Prostate cancer DTCs and drug resistant dormant myeloma cells in the bone marrow respond to osteoblast derived growth arrest specific 6 (GAS6), through Axl, a receptor tyrosine kinase and remain dormant (73, 90, 91). HSC driving factors such as osteoblast secreted stromal cell-derived factor 1 (SDF-1/CXCL12) binds to CXCR4 on cancer cells and retains them in the HSC niche (87). SDF-1 CXCR4 interaction plays an important role in keeping chronic myeloid leukemia stem cells dormant. Depletion of CXCL12 in mesenchymal stromal cells led to increased proliferation of these dormant cells while deletion of CXCL12 in endothelial cells resulted in reduced proliferation (92). Dormant breast cancer cells are predominantly found in the E-selectin and SDF-1 rich perisinusoidal vascular regions. Simultaneous blockade of CXCR4 and E-selectin in patients could release dormant micro metastases from the protective bone microenvironment and also prevent adhesion in the first place (93).

TGF-β2 rich bone microenvironment promotes quiescence in HNSCC DTCs by inducing cell cycle inhibitor p27, metastatic suppressor DEC2 and SMAD1/5 activation, while the TGF-β2 low lung microenvironment permits metastatic outgrowth. Removing this break by inhibiting TGF-βRIII increased metastatic burden in mice (94). BMP7, another TGF-β family member secreted by bone stromal cells induces senescence in prostate DTCs through BMPR2 dependent activation of p38 and p21. Withdrawal of BMP7 in this mouse model of prostate cancer induces recurrent metastatic growth in the bone (95). Similarly, BMP4 supports breast cancer dormancy in the lung, while its antagonist, Coco, drives metastatic outgrowth (96). Cancer cells have also been reported to either cannibalize bone marrow derived mesenchymal stem cells or prime them to secrete microRNA packed exosomes that promote quiescence (97, 98). WNT5a from the osteoblastic niche induces dormancy in prostate cancer cells by activating non-canonical ROR/SIAH2 signaling and repressing canonical WNT/β-catenin signaling (99). In an autocrine fashion, breast and lung cancer DTCs can also enforce a slow cycling state by inhibiting WNT/β-catenin signaling (27).

### Innate and Adaptive Leukocytes

Host immunity plays an important role in shaping and limiting tumor growth and progression (100–106). Neutrophils are the most abundant circulating immune cells and among the first ones to infiltrate the lung metastatic niche. Their role in either promoting or inhibiting metastasis is highly debated (107). MET expressing neutrophils secrete reactive oxygen species and are reported to be anti-metastatic (108, 109). In contrast, several studies identify a pro-metastatic function for neutrophils (110). Neutrophils inhibit natural killer (NK) cell function and facilitate extravasation of tumor cells by secreting IL-1β and matrix metalloproteinases (111). Neutrophil derived leukotrienes further support early colonization of breast cancer cells (112, 113). Depletion of neutrophils or genetic ablation of CXCR2, suppressed metastasis in pancreatic cancer models and lead to increased T-cell infiltration and extended survival (114). Recent reports highlight the role of neutrophils in metastatic outbreaks induced by sustained lung inflammation caused by tobacco smoke or bacterial derived lipopolysaccharide (115). Systemic inflammatory response induced after surgery can also promote the re-emergence of tumors that were kept in check by a tumor-specific T-cell response (116). Inflammation in lung, induced formation of neutrophil extracellular traps (NET) that resulted in cleavage and remodeling of laminin. Remodeled laminin activated integrin signaling and induced proliferation in otherwise dormant lung DTCs. This escape from latency is reported to be dependent on expression of Zeb1, a key modulator of EMT (117). Antibodies against NET-remodeled laminin prevented awakening of dormant cells and has been proposed as an approach to prevent metastatic outbreaks and prolong survival of cancer patients (115). Of note, obesity causes lung neutrophilia and the increase in neutrophils favors breast cancer metastasis to lung (118, 119).

Tissue resident macrophages or infiltrating monocytes are also reported to play an important role in either limiting or promoting early colonization of DTCs post extravasation (105, 120). Monocyte chemotactic and activating factor (CCL2) secreted by cancer cells and stroma recruits CXCR2^+^ positive monocytes and macrophages to enable seeding, colonization and outgrowth (121, 122). VCAM1 on breast cancer cells in leukocyte rich lung microenvironment binds to α4β1 integrin on macrophages and activates Ezrin-AKT survival pathway in cancer cells (123). In the bone, aberrant expression of VCAM1 promotes transition from indolent to overt metastasis in breast DTCs. VCAM1 expressing DTCs attract and tether to integrin α4β1 expressing osteoclast progenitors and give rise to osteolytic metastasis. Antibodies against α4 integrin block this prosurvival function of VCAM1 and metastatic burden (124). NR4A1 positive patrolling monocytes that are enriched in the microvasculature of the lung, engulf melanoma, and breast tumor cells and reduce lung colonization and metastasis (125, 126). They also promote recruitment and activation of NK cells. Administration of selective class IIa histone deacetylate (HDAC) inhibitor, in MMTV-pyMT mouse model, resulted in reduced tumor burden and spontaneous pulmonary metastasis. HDAC inhibition reverts the pro-tumorigenic phenotype of tumor associated macrophages, recruits anti-tumor phagocytic macrophages and stimulates the adaptive immune response (127). Selective inhibition of histone deacetylase may unleash the antitumor potential of macrophages and keep DTCs in check.

NK cells play an important role in surveilling and eradicating cancer cells in circulation and upon extravasation (106, 128). By releasing cytolytic granules and pro-apoptotic factors or cytokines, NK cells kill tumor cells. They also release chemokines that attract T-cells, dendritic cells, and monocytes promoting adaptive immune response (129, 130). NK cell cytotoxicity has been negatively correlated with metastatic burden in several cancer types (131, 132). Depletion of NK cells aid metastatic outbreaks in disseminated cancer cells from breast and lung cancers (27, 111, 133). As tumors become invasive and acquire mesenchymal traits, they upregulate expression of cell surface NK cell activating ligands and are more susceptible to elimination by NK cells (134). DTCs are therefore more susceptible to immune recognition in circulation and upon extravasation (106). Nonetheless, cancer cells evade NK mediated immune surveillance by either down regulating NK cell activating ligands and death inducing receptors (135, 136). For example, extravasated breast and lung cancer DTCs in lung, brain, liver, and kidneys evade immune attack by NK cells by entering into a slow cycling or quiescent state enforced by autocrine inhibition of WNT signaling pathway (27, 133). Through mechanisms yet to be defined, these slow cycling DTCs downregulate expression of several NK cell activating sensors (27).

In a spontaneous mouse model of melanoma, early dissemination of tumor cells to the lung was observed and the DTCs remained dormant for varying periods of time. Depletion of CD8^+^ T cells in these metastasis models resulted to increased metastatic out breaks (137). Similarly, depletion of CD4^+^ and CD8^+^ T cells 5 months after surgical removal of methylcholanthrene-induced fibro sarcoma tumor results in lung metastasis, highlighting the role of T cells in eliminating proliferative DTCs (138). In this model, intratumoral MHC-I heterogeneity dictates metastatic capacity and is proposed to predict response to immunotherapy (139). It is possible that the immune equilibrium at the metastatic site is maintained by the immune suppressive (MDSCs, Treg) and tumor inhibiting (T cells, NK cells) cells. Taken together, all these studies reinforce the role of innate and adaptive immune system in either delaying or limiting metastatic incidence. They also provide a framework to investigate the effect of host microenvironment on metastatic latency. Given that mouse and human immune systems are different, development of reliable preclinical models that replicate human immune surveillance are desired.

## Target Residual Disease: How to Eliminate the Veiled Threat?

Tracking residual disease in patients with no obvious symptoms is challenging. In order to accurately predict relapse, genomic and epigenomic characteristics of divergent disseminated cancer cells at the metastatic site and their associated phenotypic information is needed. Disease predictions depend on preclinical models, that are imperfect as they are based on assumptions that change with novel insights and discoveries. Nonetheless, every model, in spite of its limitations, has advanced our understanding of this phase of tumor progression (140, 141) (Table 2).

**Table 2:** Metastatic latency preclinical models.

**Cancer type**	**Preclinical model**	**Mechanistic insights into DTC biology**	**References**
Breast	HCC1954	NK cell mediated immune evasion, self-imposed quiescence, SOX9, DKK1, p-p38	(27)
	4T07	BMP signaling	(96)
	MDA-MB-231	VCAM-1 mediated osteoclastogenesis, chemoresistance by Fbxw7	(84, 124)
	MMTV-HER2	Early dissemination of DTCs, parallel evolution of metastatic cancer	(35, 36)
	PDX	Stem cell program - OCT4, SOX2, DKK1	(27, 65)
	BT549	p-p38, relieving ER stress by PERK and EIF2a	(82)
	D2A1	anti-inflammation, integrin signaling	(69, 70, 117)
Prostate	PC3	GAS6/Axl, Wnt5a signaling, BMP7	(90, 95, 99)
	DU145	GAS6/Axl	(90)
Melanoma	RET.AAD	Early dissemination of DTCs, restrained outgrowth by CD8 T-cell	(137)
Lung	H2087	NK cell mediated immune evasion, self-imposed quiescence, SOX2, DKK1	(27)
Head and neck	HEp-3	Epigenetic repression by NR2F1, low p-ERK, high p-p53/p-p38, SOX9, TGFβ2	(64, 72, 94)
Pancreas	mM1	Immune evasion by relieving ER stress	(83)
Fibrosarcoma	GR9	Low MHC-I	(138, 139)

Keeping DTCs in a quiescent non-proliferative state is an attractive viable approach to limit delayed metastatic incidence (115, 142, 143). Adjuvant anti-estrogen therapy with the ER antagonists is a standard of care for patients with ER^+^ breast cancers for years after initial diagnosis and this approach has significantly improved survival outcomes (22, 63). FDA approved CDK4/6 inhibitors for ER^+^ breast cancers, block cancer cell proliferation and induce dormancy or senescence in various models (144). Such inhibitors have potential to limit relapse in cancers with prolonged metastatic latency phase. Inhibition of integrin β1, uPAR, ERK, and Src driven signaling might prevent metastatic breakouts. Activation of p38, NR2F1, or administration of GAS6, BMP4/7, WNT inhibitors, and TGF-β2 might be effective in limiting relapse. The major challenge for this approach is identifying enforcers of quiescence that are effective in all tissues and specific for cancers with distinct oncogenomic features. Also unknown is how well tolerated these extended therapies will be in patients and how effective this approach will be on slow cycling DTCs. Nevertheless, the threat of disease relapse will still remain.

Removing the proliferative break or mobilizing DTCs from their niches and allowing anti-proliferative drugs or immune surveillance to target DTCs is an alternative strategy (83, 84, 94, 95, 145, 146). Unleashing the proliferative potential of quiescent population has disease management concerns. In order to be effective, this approach would have to drive all DTCs out of quiescence and the subsequent treatment has to effectively eliminate all proliferating cancer cells, which is unlikely. Moreover, this approach may result in selection of clones that don't respond to available therapies and can be detrimental to patient health. Eliminating quiescent DTCs by targeting intrinsic or extrinsic enforcers of this state is an attractive approach that needs to be further explored in clinic (142, 143).

## Concluding Remarks

Early detection of disseminated disease with improved understanding of cellular and molecular mechanisms driving metastatic latency in an organ with distinct tissue architecture is critical to provide effective therapeutic interventions. Designing a clinical trial to assess the benefit of proposed strategies is a major challenge. Some obvious questions apart from the cost being: how to define patients with likelihood of disease relapse, trial duration and endpoint criteria. Further research with preclinical models that faithfully represent this phase of tumor progression will provide risk prognostication tools, novel targets and treatment strategies to eliminate minimal residual disease.

## Author Contributions

SM conception, design, and wrote the manuscript. KK, MM-P, and SM literature search. All authors contributed to manuscript revision, read, and approved the submitted version.

### Conflict of Interest Statement

The authors declare that the research was conducted in the absence of any commercial or financial relationships that could be construed as a potential conflict of interest.
